# Passive acoustic monitoring of beaked whale densities in the Gulf of Mexico

**DOI:** 10.1038/srep16343

**Published:** 2015-11-12

**Authors:** John A. Hildebrand, Simone Baumann-Pickering, Kaitlin E. Frasier, Jennifer S. Trickey, Karlina P. Merkens, Sean M. Wiggins, Mark A. McDonald, Lance P. Garrison, Danielle Harris, Tiago A. Marques, Len Thomas

**Affiliations:** 1 Scripps Institution of Oceanography, University of California San Diego, La Jolla, California 92093-0205, USA; 2 WhaleAcoustics, 11430 Rist Canyon Road, Bellvue, Colorado 80512, USA; 3 National Marine Fisheries Service, Southeast Fisheries Science Center, 75 Virginia Beach Dr. Miami, Florida 33149, USA; 4 Centre for Research into Ecological and Environmental Modelling, The Observatory, University of St Andrews, St Andrews, KY16 9LZ, Scotland

## Abstract

Beaked whales are deep diving elusive animals, difficult to census with conventional visual surveys. Methods are presented for the density estimation of beaked whales, using passive acoustic monitoring data collected at sites in the Gulf of Mexico (GOM) from the period during and following the *Deepwater Horizon* oil spill (2010–2013). Beaked whale species detected include: Gervais’ (*Mesoplodon europaeus)*, Cuvier’s (*Ziphius cavirostris),* Blainville’s (*Mesoplodon densirostris*) and an unknown species of *Mesoplodon sp.* (designated as Beaked Whale Gulf — BWG). For Gervais’ and Cuvier’s beaked whales, we estimated weekly animal density using two methods, one based on the number of echolocation clicks, and another based on the detection of animal groups during 5 min time-bins. Density estimates derived from these two methods were in good general agreement. At two sites in the western GOM, Gervais’ beaked whales were present throughout the monitoring period, but Cuvier’s beaked whales were present only seasonally, with periods of low density during the summer and higher density in the winter. At an eastern GOM site, both Gervais’ and Cuvier’s beaked whales had a high density throughout the monitoring period.

Beaked whales are found in offshore waters of the Gulf of Mexico (GOM)^1^. They are challenging to study due to their pelagic habitat, the difficulty of their visual identification, and their elusive behavior, with prolonged deep dives and short surface intervals^2^,^3^. They forage on deepwater squid and fish^4^,^5^ and tend to be found in small groups of one to six animals^6^. The northern GOM, including substantial areas of beaked whale habitat, was the site of the 2010 *Deepwater Horizon* oil spill (Fig. 1). The goal of this paper is to further develop methods for density estimation for beaked whales, and potentially other odontocetes, using passive acoustic monitoring. The results of this study will be used in future efforts for modeling the potential impacts of the *Deepwater Horizon* oil spill on odontocete populations in the northern GOM.

Four species of beaked whales are known to be present in the GOM including: Cuvier’s (*Ziphius cavirostris*), Gervais’ (*Mesoplodon europaeus*), Blainville’s (*M. densirostris*) and Sowerby’s (*M. bidens*) beaked whales^1^,^4^. Cuvier’s beaked whales are distributed throughout the world’s oceans in offshore waters. A total of 18 Cuvier’s beaked whale strandings have been documented in the GOM^1^. Gervais’ beaked whales have a more narrow distribution; they are found in the warm temperate and tropical waters of the North and South Atlantic, with 16 documented Gervais’ beaked whale strandings in the GOM^1^. Blainville’s beaked whales are also distributed in temperate and tropical waters, however, they are found at low and mid-latitudes globally in the world’s oceans. There have been four documented strandings of Blainville’s beaked whales in the GOM, all confined to the northern region^1^. Sowerby’s beaked whales are thought to inhabit regions outside of the GOM, with only one stranding record there^7^; this species is more typically found in northern temperate waters of the North Atlantic.

Beaked whales produce species-specific frequency modulated (FM) upswept echolocation pulses to forage at depth (up to 1888 m)^3^ and sense their environment. Acoustic descriptions have been made for FM pulses from Blainville’s^8^,^9^,^10^, Cuvier’s^11^, Gervais’^12^, and Sowerby’s^13^ beaked whales. Because the FM pulses of beaked whales can be discriminated to species level based on their spectral and temporal characteristics, passive acoustic monitoring can provide detailed information on their daily, seasonal and geographical occurrence^14^,^15^.

Similar to other odontocetes, beaked whales concentrate their transmitted acoustic energy into a forward directed beam. For beaked whales, the beam is narrow, with an estimated directivity index (ratio of sensitivity in preferred-direction relative to omni-direction) of more than 23 dB^16^,^17^. By directing their acoustic energy into a narrow beam, the probability of detecting these animals by passive acoustic monitoring will be increased when the animals are oriented towards the acoustic receiver, and diminished when the animals are oriented away from the acoustic receiver.

Several approaches have been presented previously to estimate beaked whale densities from passive acoustic monitoring data^18^,^19^,^20^,^21^. All methods involved the estimation of the probability of detecting the object of interest (e.g., an animal, a group of animals or an acoustic cue produced by the study species), which is a key aspect of wildlife population size monitoring^22^. A cue counting approach was presented for estimating Blainville’s beaked whale densities using a hydrophone array in the Bahamas^18^, where the cues were individual echolocation clicks and detection probability was estimated using animal-borne digital acoustic tags. Another form of cue counting analysis was applied to the same species and location, using the onset of echolocation by groups of diving whales as the cue^19^. In this case, detection probability did not need to be estimated, as echolocating groups could be detected with certainty within the study area. Finally, using the same dataset, a Monte Carlo simulation was developed to estimate the probability of detecting calls as a function of distance^20^, allowing density estimation to be conducted with single hydrophone sensors.

Passive acoustic monitoring data were used to detect the presence of four species of beaked whales over more than three years, at three sites on the GOM continental slope in deep water (Fig. 1): Green Canyon (GC), Mississippi Canyon (MC), and Dry Tortugas (DT). No beaked whales were detected at two monitoring sites on the continental shelf in shallow water: Main Pass (MP) and DeSoto Canyon (DC). These continental shelf sites are presumably too shallow to be beaked whale foraging habitat. We present data on beaked whale presence first as encounters (periods of echolocation clicking lasting more than 75 sec) and then quantify them by 5-min time bins, and the number of echolocation clicks within each bin. Two methods for estimating weekly densities of beaked whales are obtained from these data, one based on the number of echolocation clicks detected (click counting) and the other based on the presence of clicks during 5-min time bins, thereby indicating the presence of animal groups (group counting). Density estimates for two species of beaked whales at the slope sites, derived from the click counting and group counting methods, revealed differences in their seasonal presence and distributions.

## Results

Four beaked whale acoustic signatures were detected in the GOM passive acoustic monitoring data (Fig. 2): Cuvier’s beaked whale, Gervais’ beaked whale, Blainville’s beaked whale, and a sound believed to be a beaked whale but of an unidentified species that we designate as “Beaked Whale Gulf ” (BWG). A key parameter used for species identification was the inter-click interval (ICI). Cuvier’s beaked whale generally had a longer ICI (510 ms) than Blainville’s beaked whale (320 ms), Gervais’ beaked whale (290 ms) or the BWG beaked whale (140 ms)^15^. The BWG beaked whale also had a longer click duration and greater sweep bandwidth than Cuvier’s, Gervais’ or Blainville’s beaked whale.

The ICIs for Cuvier’s and Gervais’ beaked whales (Supplementary Figure S1) had a strongly peaked distribution, representing a regularity of click timing during echolocation. For Gervais’ beaked whales the ICI (290 ms, CV = 0.002) was consistent between recording locations. However, the ICI for Cuvier’s beaked whales was slightly longer at the DT site (530 ms, CV = 0.003) relative to the GC site (520 ms, CV = 0.010) and the MC site (500 ms, CV = 0.011). ICI statistics for Cuvier’s, Gervais’, Blainville’s and BWG beaked whales are presented by site in Supplementary Table S1.

Beaked whale encounters were defined as instances with more than 75 seconds of clicking detected from the same species, with no more than a one-hour gap between successive clicks (Table 1). Gervais’ beaked whale was the dominant beaked whale species at the western GOM sites (68% of all beaked whale encounters at MC and 76% at GC) whereas Cuvier’s beaked whale was dominant at the DT site (61%). The BWG beaked whale was found infrequently at the western sites (4% at MC and 3% at GC) and was rarely observed at the DT site (0.1%). Blainville’s beaked whale was detected only at the GC site and only with few encounters (3%).

In the remainder of this paper we will focus on Gervais’ and Cuvier’s beaked whales for which we recorded 2,758 and 2,820 encounters respectively (Table 1). These are the dominant beaked whale species in the GOM, and those for which sufficient ancillary information was available to allow density estimation from acoustic monitoring data. Blainville’s beaked whale, for which we had few encounters, and the BWG beaked whale, for which the species is not yet determined, will not be further considered.

### Group Size

Group size estimates (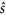) were made for Gervais’ and Cuvier’s beaked whales at each of the three deep water recording sites (DT, GC, and MC). Time-series of click-sequences for beaked whales (Supplementary Figure S2) show a regular ICI and slowly varying click amplitude, allowing the number of echolocating whales to be counted. Differences in click amplitude between the animals may represent differences in proximity to the sensor, animal orientation, or source level.

Gervais’ beaked whales were most commonly encountered as groups of two animals (Fig. 3); this pattern was observed at all sites. The mean Gervais’ group size for all sites was 2.26 (CV = 0.03) animals, with a maximum group size of 5 animals. The mean Gervais’ beaked whale group size at the DT site was slightly larger (2.80, CV = 0.08) than at the MC and GC sites (2.18, CV = 0.06 and 2.06, CV = 0.05, respectively). Cuvier’s beaked whales were more commonly encountered as single animals, with a mean group size of 1.95 (CV = 0.05) animals, and a maximum group size of 4 animals. The mean Cuvier’s group size at the GC site was slightly smaller (1.69, CV = 0.10) than at the MC site (2.10, CV = 0.09) and the DT site (1.98, CV = 0.07). Given these variations in group size, we used the measured values and error estimates by site, rather than using average values by species.

### Vocal Activity

#### Proportion of Time Clicking

The proportion of time (seconds) spent clicking during a dive cycle was determined for Cuvier’s and Gervais’ beaked whales based on acoustic tag data (Supplementary Figure S3). Due to the lack of any tag data for Gervais’ beaked whales, we used Blainville’s beaked whale data as a proxy for Gervais’ beaked whales, owing to their similarity in size (~1000 kg and ~1200 kg respectively)^4^, whereas Cuvier’s beaked whales are substantially larger (up to ~3000 kg) than Gervais’ beaked whales^4^. A dive cycle was defined to include both a deep (foraging) dive and subsequent shallow dives. For both datasets the first dive of each tag record was excluded from the analysis, as there appeared to be a tagging effect (smaller portion of dive cycle with clicks) in Cuvier’s beaked whale data (Supplementary Figure S3), but no clear effect in the Blainville’s beaked whale data. For Cuvier’s beaked whales, the mean (weighted by dive cycle length) proportion of each dive cycle that was comprised of click-positive-seconds was 0.243 (range of 0.097 to 0.380) with CV = 0.087. For Blainville’s beaked whale, the mean proportion of each dive cycle that was comprised of click-positive-seconds was 0.141 (range of 0.039 to 0.326) with CV = 0.169.

For the group counting analysis, the mean (weighted by dive cycle length) proportion of 5 min bins that contained click-positive-seconds was 0.354 (range of 0.143 to 0.67) with CV = 0.084 in the Cuvier’s dataset. In contrast, for the Blainville’s dataset, the proportion of 5 min bins across each dive cycle that contained click-positive-seconds was 0.191 (range of 0.057 to 0.438) with CV = 0.171.

#### Estimated Click Rates

Estimated click rates (*r*) were obtained from the product of the mean proportion of the dive cycle spent clicking, and the inverse of the ICI (Table S1). The click rates were remarkably similar both by site and by species. The combination of more rapid clicking by Gervais’ beaked whales (~3 clicks/s) relative to Cuvier’s beaked whales (~2 clicks/s), is compensated by a smaller proportion of time dedicated to clicking for Gervais’ beaked whales (19%) relative to Cuvier’s beaked whales (36%), yielding similar estimated click rates (~0.49 clicks/s) over their dive cycles (Table 2).

#### Vocal Synchrony

To account for group clicking behavior, records of simultaneously tracked beaked whales were examined from acoustic array data^23^,^24^. Four instances were examined in which two or more Cuvier’s beaked whales were tracked. These data revealed the time intervals for clicking of individual animals, and the proportion of overlap between clicking bouts for animals within the group. Overlap of 0.67 (CV = 0.03) was observed for clicking bouts between two Cuvier’s beaked whales. Given a 0.354 probability of clicking in a 5 min window for a single Cuvier’s beaked whale, the probability of group clicking (*P*_*v*_) in a 5 min window then becomes:


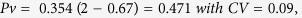


for a nominal group size of two animals in which the CV is obtained from the root of the sum of the squared CV for individual click bouts (CV = 0.08) and for overlap (CV = 0.03). The results suggests that a group of two Cuvier’s beaked whales would be vocally active about 47 percent of the time across each dive cycle. Given that the average beaked whale group size is approximately two animals, we applied a *P*_*v*_ of 0.471 for the group clicking probability (Table 3).

Since no comparable data on clicking probability are available for Gervais’ beaked whales (neither for the individual probability of clicking, nor for the group synchrony) our best alternative was an application of the probability of clicking from Blainville’s beaked whale (0.191, CV = 0.171, given above) and a synchrony estimate based on Cuvier’s beaked whales (0.67, CV = 0.03). This yields an estimate for the probability of group clicking for Gervais’ beaked whales as follows:


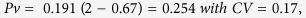


suggesting that a group of two Gervais’ beaked whales would be vocally active about 25 percent of the time across each dive cycle (Table 3).

### Detection Probability with Range

#### Click Detection

The probability of detecting beaked whale clicks as a function of horizontal range was modeled with a simulation method using the parameters described below in the Methods. The resultant detection probabilities by species (Fig. 4) were nearly identical for all sites (MC, GC, and DT) suggesting little or no dependence upon local site features. The probability of detecting clicks was estimated to be unity (all clicks detected) only for horizontal ranges of less than about 200 m for Gervais’ beaked whales, and about 400 m or less for Cuvier’s beaked whales. The detection probability fell sharply for greater horizontal ranges, owing to the highly directional beam pattern of beaked whale click production. Only the on-axis clicks were estimated to be detected beyond about one km, and the width of the beam pattern determined the probability of click detection, assuming a random orientation of the animal with respect to the sensor. The maximum horizontal range that any clicks were detected in the simulation (see Methods) was 3.2 km for Gervais’ beaked whales and 3.5 km for Cuvier’s beaked whales. Table 2 gives the estimated probability of click detection (*P*_*k*_) by species and site, adjusted for a maximum monitoring radius (*w*) of 4 km, within which about 4% (CV = 0.18) of Gervais’ beaked whale clicks and 7% (CV = 0.16) of Cuvier’s beaked whale clicks were detected.

#### Group Detection

The probability of group detection as a function of horizontal range was also modeled with the simulation method, accounting for group size and dispersion of orientation over a 5 min period (Fig. 4). The group detection probability was estimated to be unity for horizontal ranges up to 400 m for Gervais’ beaked whales and up to 600 m for Cuvier’s beaked whales; this is the range where it was likely for even an off-axis click to be received at the sensor. The detection probability was then estimated to fall to a plateau at a level of 0.7 for horizontal ranges beyond 400–600 m, and up to 2–2.5 km. The probability for these ranges was related to the variation in orientation of animals within the group, in which greater dispersion in orientation angle by group members will lead to higher probability of receiving an on-axis click from the group. The detection probability fell off rapidly beyond 2–2.5 km. Table 3 gives the estimated probability of group detection (*P*_*k*_) by species and site, adjusted for a maximum monitoring radius (*w*) of 4 km. About 28% (CV = 0.08) of Gervais’ beaked whale groups, and 36% (CV = 0.08) of Cuvier’s beaked whale groups were detected for horizontal ranges less than 4 km.

### Density of Beaked Whales

Higher densities were observed at the eastern GOM site (DT) than at the western sites (MC and GC) for both Gervais’ and Cuvier’s beaked whales (Table 2 and Table 3). Additionally, Gervais’ beaked whale densities were higher than Cuvier’s beaked whale densities at both western sites (MC and GC).

Density estimates derived from click counting and from group counting methods were in good general agreement, with a few exceptions. The two methods were in good agreement for both Gervais’ and Cuvier’s beaked whale densities at the MC and GC sites. The click counting densities for Gervais’ beaked whales were 22% and 8% lower than the densities derived from group counting at the MC and GC sites (cf. Table 2 and Table 3). Likewise, the click counting densities for Cuvier’s beaked whales were 36% and 12% lower at the MC and GC sites than the densities derived from group counting at these sites (cf. Table 2 and Table 3). The largest discrepancy was for Gervais’ beaked whales at the DT site, in which the click counting method resulted in a 50% lower estimate than that derived from group counting.

Time series of density estimates over the period from May 2010 to September 2013 are presented for Gervais’ (Fig. 5) and Cuvier’s (Fig. 6) beaked whales. At all sites, the detection of beaked whales fluctuated daily (Supplementary Figure S4 and Supplementary Figure S5), presumably as groups of animals moved in and out of the detection range of each instrument. Gervais’ beaked whales at the two western sites, MC and GC, appeared to be present throughout the monitoring period (Fig. 5). Conversely, the presence of Cuvier’s beaked whales at these sites varied seasonally (Fig. 6) with animals present primarily in the winter months (e.g. October–February). At the DT site, both Gervais’ and Cuvier’s beaked whales had a strong and consistent presence throughout the monitoring period.

## Discussion

The results presented here demonstrate that passive acoustic monitoring can be an effective means for providing a time-series of beaked whale density estimates at fixed locations. We chose to report estimates at a temporal scale of one week; the choice of time interval was a trade-off between the desire to produce estimates at as fine a scale as possible, for subsequent analyses (see below), and the requirement for a reasonably long time interval so that our assumptions about animal positions with respect to the hydrophone (see Monte Carlo simulations, in the Methods) be met. A key motivation for estimating density on a weekly time scale was to provide information on temporal trends such as might occur with animal movements or changes in population. In addition, the weekly estimates obtained here will be used as input to future environmental models, where oceanographic parameters (e.g. sea-surface temperature) may also vary on a comparable time scale. However, we do not anticipate that inferences drawn from subsequent analyses will be very sensitive to choice of time period within a reasonable range (e.g., weekly, bi-weekly, monthly), particularly since longer time intervals will produce fewer estimates for subsequent analyses (e.g., trend analysis) but with lower temporal autocorrelation. The ability to produce a nearly continuous time series of density estimates is a key advantage of fixed passive acoustic monitoring over current visual methods, where it is not possible to collect comparable numbers of detections nor to collect data over a continuous and long-term time period (e.g. 3 years).

Passive acoustic monitoring at a few static sites, as presented here, is limited in its ability to determine absolute population abundance, owing to restricted spatial coverage. Rather, densities specific to the monitoring sites are presented (Table 2 and Table 3), and the extent to which these sites represent a larger area is uncertain.

There are several aspects of beaked whale behavior that make them amenable to density estimation using passive acoustic monitoring of their echolocation clicks. First is their deep foraging behavior, which requires that they are dependent upon echolocation for prey detection^2^,^3^. Their use of echolocation is primarily restricted to the deeper portions of their dives, and it is conducted with a nearly metronomic ICI. These factors aid in the reliability of using an average click rate to represent their behavior when averaged across their dive cycle. Additionally, beaked whales are generally found in small groups of animals when foraging, making it easier to discern the echolocation clicks of individual animals (Supplementary Figure S2) and to determine the average group size using acoustic methods (Fig. 3).

Recent technical advances have allowed for detailed study of beaked whale behavior, turning them from one of the most poorly known species to one of the better known with regards to echolocation behavior. Digital acoustic tags have documented beaked whale diving and echolocation behaviors with sufficient detail to allow estimation of average click rates (Supplementary Figure S3). Likewise, advances in acoustic tracking have allowed for estimation of the source level and beam pattern for echolocation clicks, and monitoring of multiple animals within a diving group has allowed for estimation of the degree of synchrony of their echolocation bouts. These parameters have all been estimated for Cuvier’s beaked whale (Table 2 and Table 3); what is needed is both more of these data to provide better statistics and for the full range of beaked whale species to have at least the same level of data collection as has been accomplished for Cuvier’s beaked whale.

### Detection Probability Estimation

Density estimation using distance sampling-based methods^21^ requires that we have modeled adequately the detection probability of the animals with respect to their horizontal range from the acoustic sensor. We have used a simulation approach to modeling detection probability that assumes we have accurate estimates for several parameters. One potential source of error in the detection probability estimation is due to the contribution of the beam pattern inherent in beaked whale echolocation. It is thought that beaked whale clicks have a directionality index of 23 dB or more^16^,^17^, making them much more likely to be detected when the animal is directly oriented toward the sensor. In the click counting method the width of the beam pattern and the off-axis source level directly contributed to the overall detection probability. Beyond a few hundred meters horizontal range, only clicks that were nearly on-axis were detected, making the overall detection probability low (4–7% for maximum horizontal range of 4 km). The contribution of echolocation directionality to the group counting method is reduced, because the animals most likely will vary their orientation during a 5 min time window, and all the animals in a group are unlikely to remain aligned. Modeling the group detection probability, however, requires knowledge of group behavior including the rate at which animals change their heading as well as how likely the group of animals is to have dispersed orientations. These parameters might be estimated from beaked whale foraging behavior in which animals have been observed to change their orientation during bouts of clicking^25^. However, we have little data on the behavior of groups of two or more animals, and our understanding is currently based on small sample sizes.

In general, the simulation approach for detection probability requires a characterization of the detection process that was applied to the data. We have removed encounters of less than 75 s duration, and 5 min time-bins with less than 5 click detections, in order to minimize the number of false detections. However, it is possible that some valid encounters and time-bins were eliminated from the dataset by this procedure. Our current approach for modeling detection probability does not account for these missed data values, although it might be possible to incorporate these constraints into future modeling efforts. In addition, the simulation model assumes a distribution of animals placed uniformly around the sensors, which is an assumption that should be tested by tracking the locations of the animals.

After detecting clicks using an automated algorithm, we removed false encounters and time bins with manual editing. False detections were typically caused by the presence of ships or other odontocetes (sperm whales and dolphins). Misclassified encounters were also manually reclassified to the correct species; misclassification did not occur due to click amplitude but rather due to their spectra and timing, and therefore had little or nothing to do with the detection threshold. Consequently, bias on the number of clicks or bins containing clicks counted due to species misclassification could not be accounted for by the probability of detection, and so this separate reclassification process was required.

Another set of potential errors results from the need to know echolocation source levels. The received amplitude of the click is related to the source level and the range at which the click is detected. Higher amplitude clicks will be received above the detection threshold (121 dB pp: re 1 μPa) at greater ranges than lower amplitude clicks. Therefore, it is critical that the source level be known to estimate the detection range. For Cuvier’s beaked whale there is a single published estimate of on-axis echolocation click amplitude (214 dB pp re: 1 μPa), based on signals received from two simultaneously tagged animals in the Mediterranean Sea^16^, however, this value is a lower bound due to clipping of the receiving array. We supplemented these data with acoustically tracked Cuvier’s beaked whales from offshore southern California, where source levels of 225 dB pp re: 1 μPa were observed^24^. It would be more satisfying to have data for Cuvier’s beaked whale source levels in the GOM. For Gervais’ beaked whales, no measurements of source level have been made, so there is greater uncertainty of their source level and beam pattern. The Gervais’ beaked whale source level estimate that we have used (220 dB pp re: 1 μPa) could be inaccurate, leading to biased detection probabilities and therefore biased density estimates. Likewise, greater than anticipated variability in source level would result in potential greater uncertainty in the detection probability error estimate.

### Group Size and Clicking Rates

We have used overlapping click sequences to estimate the number of beaked whales in a group of presumed interacting animals. This process requires all the animals in the group to be producing click sequences and be detected at the same time. This may not be the case for young animals; it is unknown at what age beaked whales first begin to undertake foraging dives and produce echolocation signals at depth. Therefore, our method may have underestimated the contribution of young animals to the group. Likewise, if the groups were widely dispersed, our method would miss animals that remained too far from the sensor to be detected. Both non-clicking animals in the group and missed animals in the group would result in an under-estimate of the average group size and therefore the density.

Clicking rates were estimated from Cuvier’s and Blainville’s beaked whale tag data obtained in the Mediterranean and the Bahamas, and also from Cuvier’s beaked whale acoustic tracking data obtained off southern California. It would reduce potential bias and uncertainty if these data had been obtained from beaked whales in the GOM and for both of the species being investigated, and if a greater number of encounters were available to better estimate their variability.

## Conclusions

Passive acoustic data were analyzed to estimate the density of beaked whales at three sites in the GOM during and following the *Deepwater Horizon* oil spill. The two dominant species of beaked whales in the GOM were found to be Gervais’ and Cuvier’s beaked whale. Also less frequently encountered were Blainville’s beaked whale, and an as yet unidentified species of beaked whale (BWG) that may be visually similar to one of the other species. Densities of Gervais’ and Cuvier’s beaked whales were estimated based on both click and group counting methods. Potential errors in these density estimates relate to detection probability, group size and vocalization rates. Future efforts will be directed at modeling the potential impacts of the *Deepwater Horizon* oil spill on beaked whale populations in the northern GOM by comparing long-term time series of animal density with the presence of oil and other environmental parameters.

Generalizing these methods to other odontocetes, and indeed to non-marine echolocating species, presents some challenges. Delphinids, for instance, may have a strong diel pattern to their echolocation, in some instances foraging primarily at night. These diel patterns, therefore, must be accounted for in the density estimation methods applied to these species. Additionally, when echolocating animals are present in large groups, it is not clear that the total number of clicks produced is a simple linear function of the number of animals present. In this case, a group size dependent click rate may be required. Also, the diving and echolocation behavior of some odontocetes is largely asynchronous, so an accurate estimate of the degree of synchrony is needed to estimate the group detection probability. These are some of the challenges that await solution for the application of passive acoustic monitoring to a broader range of echolocating species.

## Methods

**Figure 1:**
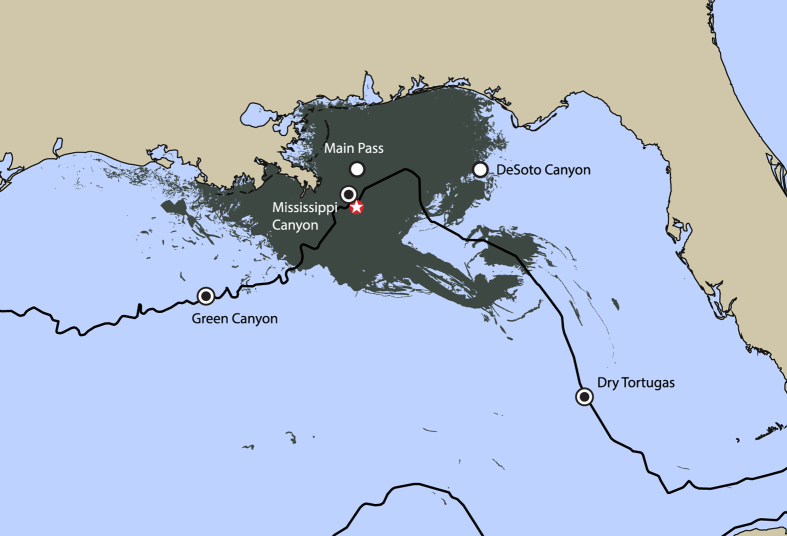
Three sites in the Gulf of Mexico with detections of beaked whales (dots): Green Canyon (GC), Mississippi Canyon (MC), and Dry Tortugas (DT); and two sites with no beaked whale detections (open circles): Main Pass (MP) and DeSoto Canyon (DC). Deepwater Horizon site (red star) and cumulative surface oil during April-August 2010 (dark gray area). The black line denotes the 1000 m contour. Surface oil is cumulative NESDIS SAR composite from: http://gomex.erma.noaa.gov. Map generated using GMT (http://gmt.soest.hawaii.edu/projects/gmt).

**Figure 2:**
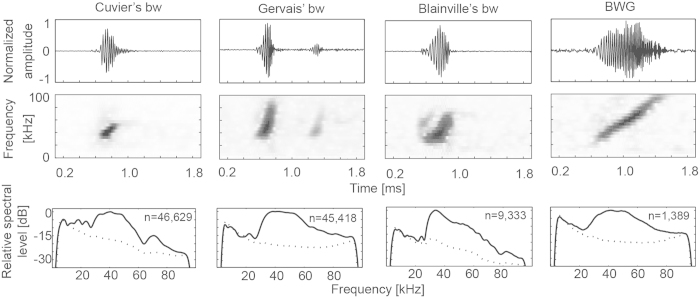
Acoustic signatures for GOM beaked whales. Time series (upper), spectrogram (middle) and mean spectra (lower; n indicates the number of FM pulses used for calculation of mean) are presented for Cuvier’s, Gervais’, Blainville’s, as well as an unknown species designated as the “BWG” beaked whale. Dotted lines show mean noise floor.

**Figure 3:**
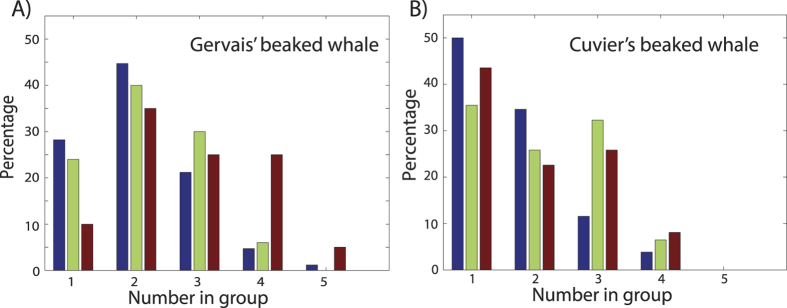
Group size distribution for (**A**) Gervais’ and (**B**) Cuvier’s beaked whales derived from acoustic encounters at MC (green), GC (blue), and DT (red) sites. In each plot the bars of each color add to 100%.

**Figure 4:**
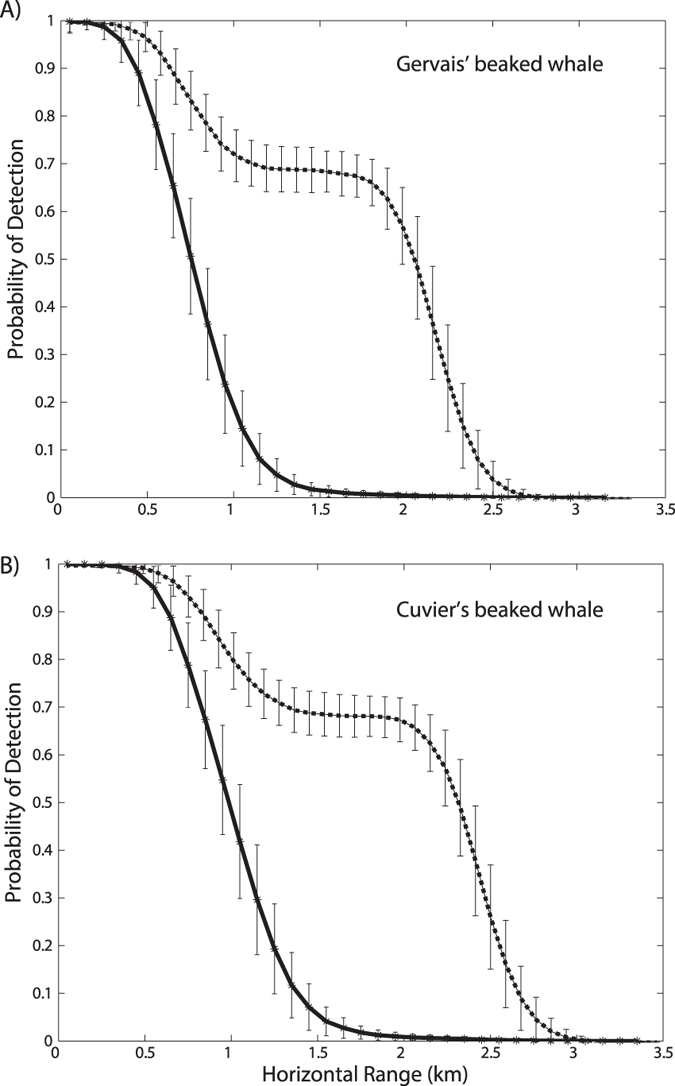
Estimated detection probability for (**A**) Gervais’ and (**B**) Cuvier’s beaked whale clicks (line) and group (dash) based on a simulation using sound propagation modeling for site MC. Only MC is shown due to the similarity of other sites. Vertical bars show +/− one standard deviation.

**Figure 5:**
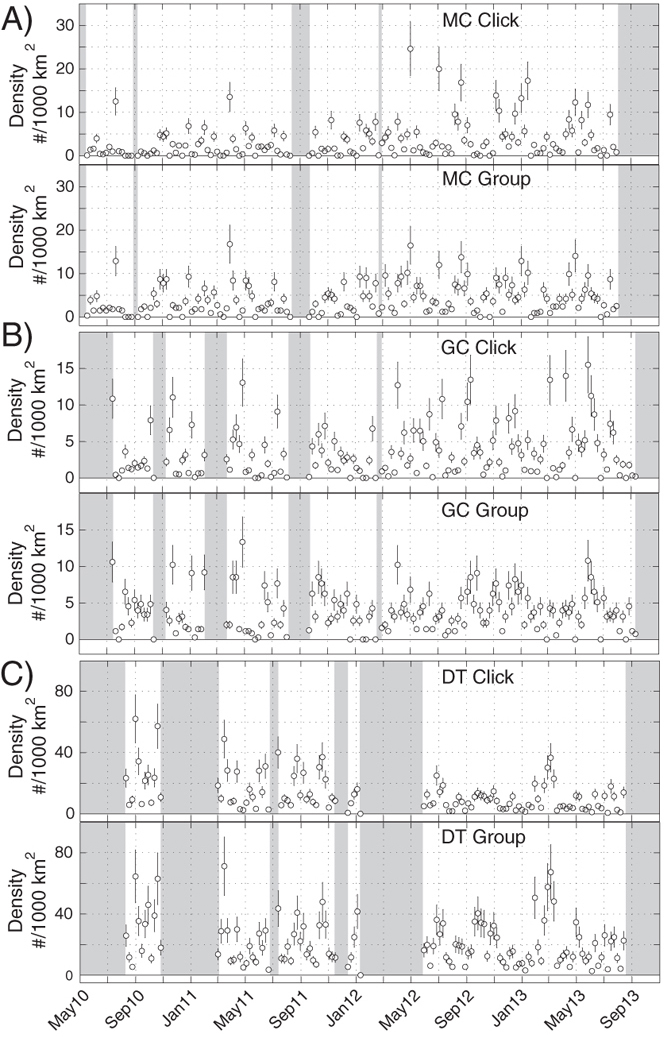
Weekly density estimate for Gervais’ beaked whale at (**A**) MC, (**B**) GC, and (**C**) DT sites based on click counting (above) and group counting (below). Circles denote estimates and vertical lines show +/− one standard error. Shaded areas lack recording effort.

**Figure 6:**
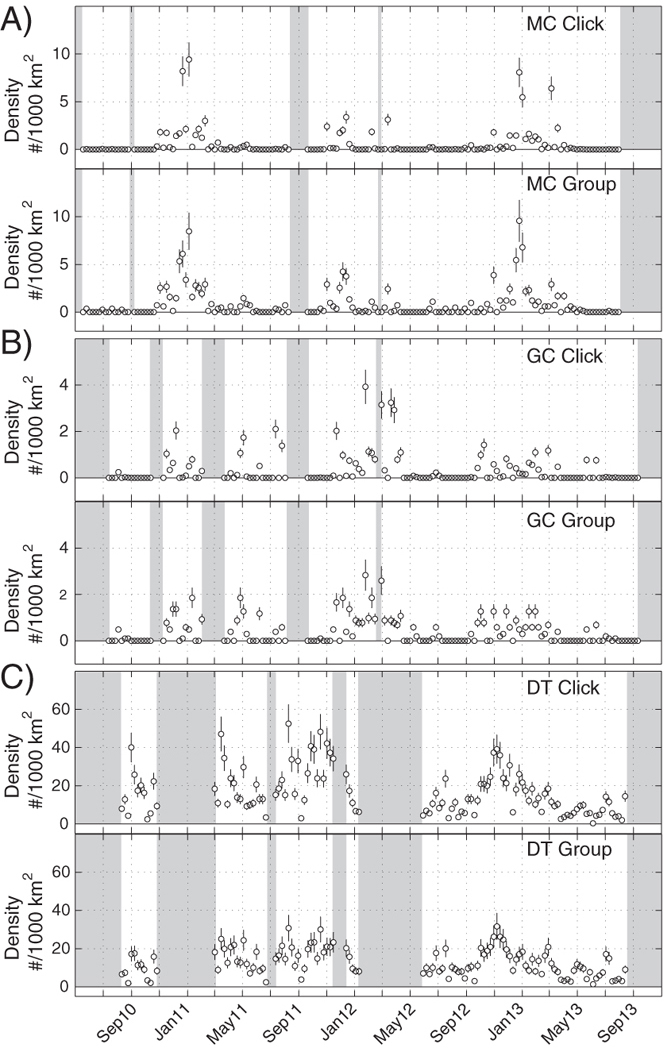
Weekly density estimate for Cuvier’s beaked whale at (**A**) MC, (**B**) GC, and (**C**) DT sites based on click counting (above) and group counting (below). Circles denote estimates and vertical lines show +/− one standard deviation. Shaded areas lack recording effort.

The data presented here were collected from three deepwater locations in the GOM (Fig. 1). All sites were named based on the federal lease block in which they are located: Green Canyon (GC), Mississippi Canyon (MC), and Dry Tortugas (DT). At each site a High-frequency Acoustic Recording Package (HARP) was deployed and recorded continuously at 200 kHz for 2–9 months per deployment during 2010–2013. Details of each deepwater HARP deployment are presented in Supplementary Table S2.

HARPs are bottom-mounted acoustic recorders containing a hydrophone, data logger, battery power supply, ballast weight, acoustic release system, and flotation^26^. The hydrophone was tethered to the instrument and buoyed approximately 10 m above the seafloor. All acoustic data were converted to sound pressure levels based on hydrophone calibrations performed at Scripps Institution of Oceanography and the U.S. Navy’s Transducer Evaluation Center facility in San Diego, California.

### Signal Description, Detection and Classification

Trained analysts identified beaked whale FM echolocation pulses in the acoustic data by first running a Teager energy click detector^27^ and beaked whale expert system (using pulse parameters), and then manually verifying each detected beaked whale click bout using custom software for analyst-assisted species classification^15^. Beaked whale signals have a longer duration than delphinid clicks, a stable ICI, and an upswept frequency modulation. When beaked whale echolocation signals were noted the sequence was inspected more closely to determine the inter-click-interval (ICI), and the presence or absence of FM pulses was determined by examining the time series and spectrogram (Hann window, 60 samples/3.3 kHz bandwidth, 98% overlap) of 3 ms time segments. Analysts initially labelled these acoustic encounters as having been produced by either one of the species whose echolocation signals are well known, or by one of the echolocation signal categories of undetermined origin^15^.

All beaked whale acoustic encounters were reviewed in a second analysis stage to remove false detections, correct misidentified beaked whale clicks, and provide a consistent detection threshold. Individual echolocation signals were automatically detected using a computer algorithm during time periods when FM pulses were manually verified using the procedure described above. FM pulse detections occurred when the signal in a 10–100 kHz band exceeded a detection threshold of 121 dB pp re: 1 μPa. The acoustic encounters were then manually reviewed using comparative panels showing long-term spectral average, received level, and inter-pulse interval of individual clicks over time, as well as spectral and waveform plots of selected individual signals. Encounters of less than 75 s duration were discarded to minimize the number of false detections. Within each encounter, periods with false detections were removed by manual editing, for instance, when the detections were identified as being from vessels, sonars, sperm whales or delphinids, owing to inappropriate spectral amplitude, ICI, or waveform. In addition, this step provided a check on beaked whale species classification, and misidentified encounters were corrected. The entire dataset was examined in this way three times. Following each examination, false detections were removed and misidentified detections were re-assigned; the process was terminated after the third iteration at which time all encounters had been manually verified and the false detection rate for encounters was determined to be zero. We further examined 10,000 randomly selected clicks and found an average false detection rate for individual clicks of 5.9% (CV = 0.04). These data were further divided into 5 min time-bins, and 6000 time-bins were randomly selected for examination; an overall false detection rate of 0.5% (CV = 0.17) for entire 5 min time-bins was found. The most common signals that were falsely identified as beaked whale clicks were delphinid and sperm whale clicks.

The next step was to determine the number of detections per unit effort. We examined these data both as the number of detected clicks, and as the number of 5 min time-bins with at least 5 clicks. We first collated the effort data and the count data by creating time bins of one week over the on-effort periods at each site. Then we associated detection counts and 5 min bin counts with each one of these weeks. A one-week period was chosen to provide a sufficient number of 5 min time-bins (2,016) for density estimation. Of the 2,016 time bins that were examined each week, the mean number of bins (averaged across sites) that contained clicks was 3–80, and these had a mean of 300–12,000 beaked whale clicks.

### Density Estimation

The goal of the analysis was to provide a density estimate for beaked whale species by site at the finest temporal resolution possible. To help ensure that the assumed conditions regarding detection probability were met for each estimate, density estimates were reported for weekly periods. At the finest temporal scale, we determined the animals’ presence and the number of detected clicks during each 5 min time period for which we had data, and then averaged over a weekly time interval. We estimated animal density using distance sampling-based methods^21^ with both time-bin detection (group-based) and click detection (cue-based) approaches.

#### Group Counting

A group counting approach for density estimation requires detection of the presence of animals within a set of short time windows, along with knowledge of the detectability of clicks produced by the group. It further relies on knowledge of both the mean group size and group vocalization behavior. Using a group counting approach, the estimated density *D*_*kt*_ at site *k*, during week *t* is:


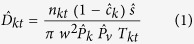


where *n*_*kt*_ represents the number of time intervals (5 min windows) that groups were detected at site *k* during week *t*, and *T*_*kt*_ represents the total number of time intervals that were sampled at site *k* during week *t*. The probability of detecting a group within a horizontal radius of size *w* (beyond which no detections are assumed possible) is *P*_*k*_, and the probability of a group being vocally active in a 5 min window is ^*P*^*v*. To account for an imperfect detection process *c*_*k*_ is the proportion of false detections. Weeks were taken as Sunday through Saturday for all sites, and at the beginning and end of deployment periods, at least two days of data were required to produce a weekly estimate, otherwise data were associated with the adjacent weekly estimate. Varying effort across weekly estimates was accounted for by *T*_*kt*_ in equation 1.

The variance was obtained using the delta method approximation^18^:


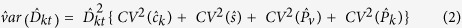


where *CV(x)* denotes the coefficient of variation of the random quantity *x*, (i.e., the standard error of the estimate of *x* divided by the estimate). Confidence intervals were obtained from the estimated variance by assuming that density follows a log-normal distribution to preclude negative values^18^.

#### Click Counting

A cue-based approach for density estimation requires counting the number of detected clicks, along with knowledge of the click production rate for individual animals and the detectability of individual clicks. Given *n*_*kt*_ detected cues (echolocation clicks) in a time period *T*_*kt*_ (sum of time periods at site *k* during week *t*), density *D*_*kt*_ can be estimated by:


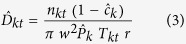


where *P*_*k*_ is the probability of detecting a vocal cue that is produced within the radius *w* from the site *k, c*_*k*_ is the proportion of false detections, and *r* is the cue production rate. Variance is obtained using the delta method approximation, as given in equation 2.

### Group Size

Estimates of beaked whale group size were derived from acoustic encounters based on overlapping click sequences with consistent ICIs. We selected beaked whale encounters with high received amplitude, suggesting that the animals were located near to the acoustic sensor. Then, we estimated the number of echolocating animals in the group by counting the number of overlaying sequences in the time series, looking for amplitude changes and consistent ICIs (Supplementary Figure S2). The basic assumption of this approach is that all animals in a group vocalize and are detected simultaneously at least at some point, so we can use the number of overlapping sequences as an estimate for group size. Each animal within the group, over a short time period, will produce echolocation clicks at a consistent ICI, and from one click to the next there is a relative consistency of amplitude, given that several clicks are produced per second and the distance and orientation of the animal will not change substantially from one click to the next. We assume that the sample collected by this method is representative of the group sizes present in the population.

### Vocal Activity

Both the click counting and group counting methods require multipliers related to vocal activity to estimate beaked whale density: the click-based method requires an estimate of mean click production rate (*r*), while the group-based method requires the proportion of 5-min bins a group is vocally active (*P*_*v*_).

Ideally, these data would be obtained from the animals being studied, at the same time and place as the acoustic survey. However, due to a lack of auxiliary data for beaked whales in the GOM, the click rates used here were derived from a combination of beaked whale acoustic tag data^28^ collected at a variety of study sites and from data on the ICI estimated from the acoustic recordings in this study. The tag records were used to estimate the proportion of seconds containing clicks produced by the tagged animal during the tag deployment duration. The ICI data were then used to predict the number of clicks produced during these “click-positive-seconds”. The combination of these two values allowed estimation of the click production rate for each species. This was done separately for each site, due to the site-specific estimates of the ICI.

The above approach for click production rate estimation allowed data from the acoustic recordings to be used where possible. Animals in the GOM may have a different ICI from the tagged animals (from the Mediterranean in the case of the Cuvier’s beaked whales and see the discussion below regarding Gervais’ beaked whales). Using ICI data from the GOM (Supplementary Figure S1) meant that it did not have to be assumed that the tagged whales and the GOM whales had the same ICIs, however, all other aspects of their diving and clicking behavior were assumed to be the same.

#### Proportion of Time Clicking

The proportion of time spent clicking, and therefore potentially detected by passive acoustic monitoring, was estimated from tag data. A Cuvier’s beaked whale estimate of click-positive-seconds was obtained from an analysis of 12 digital acoustic recording tag deployments in the Mediterranean Sea (M. Johnson *pers. comm*). These tags contained a varying number of dives, which were then split into dive cycles; a dive cycle was defined as one deep dive plus associated shallow dives. For each tag, any second containing a click was marked, and the number of click-positive-seconds was calculated for each dive cycle. This analysis accounted for times when the animal was producing clicks at a regular rate and accounts for silent times, such as when the animal was at the surface, or during a foraging dive when there was a pause in regular clicking, for instance, when buzz clicks were produced.

Gervais’ beaked whale click rates were obtained from a separate analysis of tag data. An estimate of Blainville’s beaked whale click-positive-seconds from six tag records obtained in the Bahamas^18^ was used as a proxy for Gervais’ beaked whale click rates, as no tag data exists for this species. Using the procedure described above, the mean proportion of each Blainville’s beaked whale dive cycle that was comprised of click-positive-seconds was estimated.

For both datasets the first dive of each tag record was excluded from the analysis, as there appeared to be a tagging effect (smaller portion of dive cycle with clicks) in the Cuvier’s beaked whale data but no clear effect in the Blainville’s beaked whale data (Supplementary Figure S3). To be conservative, all first dives were excluded from both datasets.

Clicking proportions for 5 min bins were calculated in the same way. Each dive cycle (excluding the first) was split into 5 min time periods and the portion of these bins with clicks was estimated. A random start point was defined for each dive cycle and the dive cycle was made cyclical (i.e., the end was conceptually joined to the beginning). Using the start point as the first second, the whole dive cycle was split into 5 min periods, and the last incomplete 5 min bin was excluded from the analysis. This randomization of the start point removed any effect of having the same start point on the proportion of 5 min bins spent clicking. The randomization also meant that discarding the last incomplete bin should not bias the mean estimate.

#### Inter-Click Interval Estimation

To estimate the ICI for each species, detected clicks were examined for every deployment. In addition to the regular clicking from a single animal, these data included clicks of multiple individuals and also measurements between clicks where an intermediate click might have gone undetected. Due to the presence of these large ICI values, it was determined that the mode of the distribution was the most robust way of estimating the ICI for each species.

The data were binned into the same weekly bins as used for the detection data. Not all weekly bins contained ICI data, but for those with 100 or more ICI data points (with values less than one second), a modal value was estimated by using a non-parametric kernel density smoothing algorithm on ICI values between 0 and 1 s. These modal values were converted to click rates by using the reciprocal. The weekly bins had varying lengths, so the weighted mean of the click rates was estimated using the weekly bin length as the weight. The variance of the weighted mean was then estimated using Cochran’s formula^29^,^30^, and the CV was also estimated.

#### Estimated Click Rates

For each species, the mean proportion of click-positive-seconds was multiplied by the inverse of the ICI (number of clicks produced per second) to give an estimated average click production rate. This was done separately for each site, due to the site-specific estimates of the ICI. The CV for the click rates was obtained from a combination of two independent sources of uncertainty, one from the proportion of time spent clicking and the other from the ICI, which were both inserted into the variance estimator using the delta method.

#### Vocal Synchrony

To estimate the probability of detecting vocal activity from an entire group of animals, the synchrony of their echolocation clicking is an important parameter. When animals in the group dive and click in asynchronous bouts, the probability of detecting the group as a whole increases. The probability of a group being vocally active in any given 5 min period increases with group size if asynchrony is present. Cuvier’s beaked whale group clicking synchrony was estimated from the timing of click bouts obtained from acoustic tracking arrays^23^,^24^. The average percent overlap between the click bouts of two or more animals was noted for encounters with Cuvier’s beaked whale tracked in deepwater regions off southern California. Encounters were selected for study when the start and end time of clicking from multiple animals could be clearly seen. From these data the following relation is used to derive the probability of group vocal activity (*P*_*v*_):


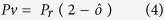


where *P*_*r*_ is the probability of vocal activity by an individual animal, and a group size of two animals is assumed. The overlap *o* is defined as the mean period when at least two animals are vocalizing, divided by the mean period of vocalization for each individual animal. It is understood that the maximum allowed value of *P*_*v*_ is unity (the probability that at least one member of the group will be vocalizing at all times). The variance of *P*_*v*_ is obtained by treating each bout of vocalizations from a group of animals as a sample unit.

Estimates of Gervais’ beaked whale group vocal activity were approximated using the same factor as obtained for Cuvier’s beaked whale. Since no acoustic tracking data exist for Gervais’ beaked whales, Cuvier’s beaked whales were selected as the best available proxy.

### Detection Probability

Knowledge of the detection probability as a function of horizontal range is needed to estimate the area that is being effectively monitored, which enables density to be estimated^18^. We used a Monte Carlo simulation approach similar to Kusel, *et al.*^20^ that is based on the source level, propagation loss, and orientation behavior of the animals. We estimated the detection probability both for single echolocation clicks and for groups of echolocating animals.

The sonar equation relates the click received-level (*RL*) to the range (*r* in meters), and the peak frequency (*f*_*p*_), including both the frequency dependent attenuation (α(*f*_*p*_)) and spherical spreading of the signal, as follows:


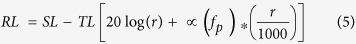


where a source level (SL) of 225 ± 3 dB pp re: 1 μPa @ 1 m was used for Cuvier’s beaked whales and a SL of 220 ± 3 dB pp re: 1 μPa @ 1 m was used for Gervais’ beaked whales. Although the source level for Gervais’ beaked whales has not been measured directly, based on their smaller body size we assume that it is less than the value for Cuvier’s beaked whales, suggesting a reduced signal level for a given range. Using peak frequencies at 40.2 kHz for Cuvier’s beaked whales and 43.8 kHz for Gervais’ beaked whales^15^, and absorption of sound in seawater^31^ with salinity S = 35 ppt, temperature T = 6 °C, acidity pH = 8 and depth = 0.98 km, provides attenuations of α(40.2 kHz) = 10.05 dB/km and α(43.8 kHz) = 11.30 dB/km for Cuvier’s and Gervais’ beaked whale click sound absorption rates, respectively. We further assume that both beaked whale species have mean dive altitudes of 175–225 m (height above the seafloor, based on tag data) and that the acoustic receiver is 10 m above the seafloor^23^. Under these assumptions, a received level of 155 dB pp re: 1 μPa corresponds to Cuvier’s beaked whales at 1 km range from the sensor, and 149 dB pp re: 1 μPa corresponds to Gervais’ beaked whales at 1 km range from the sensor.

#### Monte Carlo Simulations

Simulations that predicted beaked whale detection probability as a function of horizontal range were developed for the click based and the group based methods by combining information on animal behavior and environmental sound propagation. The first model calculated the detection probability for a single echolocation click as a function of range from the acoustic sensor. Estimated parameters for source level and mean dive altitude above the seafloor, as described above, were used. In addition, we assumed that echolocation click directivity was 24–28 dB pp (@ 40 kHz) based on beam pattern data for Cuvier’s beaked whales tracked in the Mediterranean^16^ and off southern California^24^. We assumed a mean body angle of 0 degrees (SD = 5–15 degree) from the horizontal (body axis parallel to the seafloor) during the foraging portions of the dive. Beam pattern was assumed to be radially symmetric, so roll angle was not included in the model. All headings were given equal probability (a uniform distribution in the circle) for each modelled click, since we do not have data on direction of travel at our sites. Transmission loss was simulated using the ray tracing algorithm Bellhop^32^, with inputs including bathymetry, sediment composition, sound speed profile, and mean surface roughness from the Oceanographic and Atmospheric Master Library (OAML). A model run involved placement of 10,000 echolocating animals within a 4 km radius of the acoustic sensor, with random azimuthal orientation and sound production parameters as described above. The click was designated as being detected if its received level was at or above 121 dB pp re: 1 μPa (the detection threshold used in the signal analysis). A total of 500 model iterations were run, and the mean probability for click detection was derived from the mean of these 500 iterations, as was the variance.

For simulating the probability of detecting echolocation from a group of animals, the orientation of the group during a 5 min time-bin was allowed to vary. We assumed 140–160 degrees of azimuthal rotation, and 55–65 degrees of elevation rotation while foraging or 10–15 degrees of elevation rotation while descending. The additional freedom of orientation made it more likely that an on-axis click was received from the group during each 5 min time-bin than would be expected for single clicks from individual animals. We further assumed that the highest amplitude click was produced at the center location of the group, and that the spread of group members relative to the center location of the group was small. For each species and site, 500 iterations were implemented with 10,000 groups per iteration. Averaging these data in 100 m range bins resulted in an estimate of the group detection probability.

## Additional Information

**How to cite this article**: Hildebrand, J. A. *et al.* Passive acoustic monitoring of beaked whale densities in the Gulf of Mexico. *Sci. Rep.* **5**, 16343; doi: 10.1038/srep16343 (2015).

## Supplementary Material

Supplementary Information

## Figures and Tables

**Table 1:** Beaked whale encounters by site and species with effort given in Supplementary Table S2.

Site	Total Encounters	Zc		Me		Md		BWG	
		#	%	#	%	#	%	#	%
MC	939	261	28	637	68	0	0	41	4
GC	773	144	19	587	76	22	3	20	3
DT	3953	2415	61	1534	39	0	0	4	0.1

Zc = Cuvier’s, Me = Gervais’, Md = Blainville’s, and BWG = unidentified species of Beaked Whale in the Gulf.

**Table 2:** Average beaked whale densities derived from click counting by site (MC, GC, DT) given in # of animals per 1000 km^2^.

Species	Site	Density (#/1000 km^2^) ± st dev		n_kt_/T_kt_ (#/sec)	c_k_ (% False clicks)	CV	r Click Rate (#/sec)	CV	w Max Range (km)	P_k_ Prob Detect	CV
Gervais	MC	3.29	±0.82	0.00377	7.3	0.04	0.492	0.169	4.0	0.043	0.180
	GC	3.45	±0.86	0.00385	6.4	0.04	0.484	0.169	4.0	0.043	0.178
	DT	12.60	±3.18	0.01431	4.9	0.04	0.488	0.169	4.0	0.044	0.183
Cuviers	MC	0.57	±0.11	0.00105	6.0	0.04	0.493	0.088	4.0	0.070	0.160
	GC	0.33	±0.06	0.00057	5.6	0.04	0.470	0.088	4.0	0.069	0.158
	DT	16.12	±3.05	0.02744	5.5	0.04	0.457	0.087	4.0	0.070	0.163

Parameters used for density estimation include the average number of clicks per second n_kt_/T_kt,_ the percentage of false clicks c_k_ with associated CV, the expected click rate r with associated CV, the maximum horizontal detection range w, and the probability of click detection P_k_ with associated CV.

**Table 3:** Average beaked whale densities derived from group counting by site (MC, GC, DT) given in # of animals per 1000 km^2^.

Species	Site	Density (#/1000 km^2^) ± st dev		n_kt_/T_kt_ (# bins/total bins)	c_k_ (%False bins)	CV	S Group Size	CV	P_v_ Prob Group Vocal	CV	w Max Range (km)	P_k_ Prob Detect	CV
Gervais	MC	4.09	±1.07	0.00677	0.7	0.17	2.18	0.06	0.254	0.17	4.0	0.281	0.085
	GC	3.75	±0.97	0.00655	0.3	0.17	2.06	0.05	0.254	0.17	4.0	0.281	0.082
	DT	20.96	±5.61	0.02670	0.5	0.17	2.80	0.08	0.254	0.17	4.0	0.278	0.086
Cuviers	MC	0.82	±0.19	0.00335	1.3	0.17	2.10	0.09	0.471	0.09	4.0	0.359	0.078
	GC	0.37	±0.09	0.00189	0.8	0.17	1.69	0.10	0.471	0.09	4.0	0.360	0.081
	DT	12.67	±2.78	0.05440	0.3	0.17	1.98	0.07	0.471	0.09	4.0	0.358	0.078

Parameters used for density estimation include the average number of 5 min bins with detected groups n_kt_/T_kt_, the percentage of 5 min bins with false detections c_k_ with associated CV, the expected group size S with associated CV, the probability of group vocal activity P_v_ with associated CV, the maximum horizontal detection range w, and the probability of group detection P_k_ with associated CV.
