# Supplementary material for: Passive acoustic monitoring of beaked whale densities in the Gulf of Mexico
Source: Sci Rep. 2015 Nov 12;5:16343. doi: 10.1038/srep16343 (PMC4642294; doi:10.1038/srep16343)
Supplement: Supplementary Information [file srep16343-s1.doc]

# Supplementary Information

# Passive acoustic monitoring of beaked whale densities

# in the Gulf of Mexico

John A. Hildebrand*, Simone Baumann-Pickering, Kaitlin E. Frasier, Jennifer Tricky,

Karlina P. Merkens, and Sean M. Wiggins

Scripps Institution of Oceanography, University of California San Diego,

La Jolla, California 92093-0 205, USA

Mark A. McDonald

WhaleAcoustics, 11430 Rist Canyon Road, Bellvue, Colorado 80512, USA

Lance P. Garrison

National Marine Fisheries Service, Southeast Fisheries Science Center, 75 Virginia Beach Dr. Miami, Florida 33149, USA

Danielle Harris, Tiago A. Marques, and Len Thomas

Centre for Research into Ecological and Environmental Modelling

The Observatory, University of St Andrews, St Andrews, KY16 9LZ, Scotland

## Supplementary Information


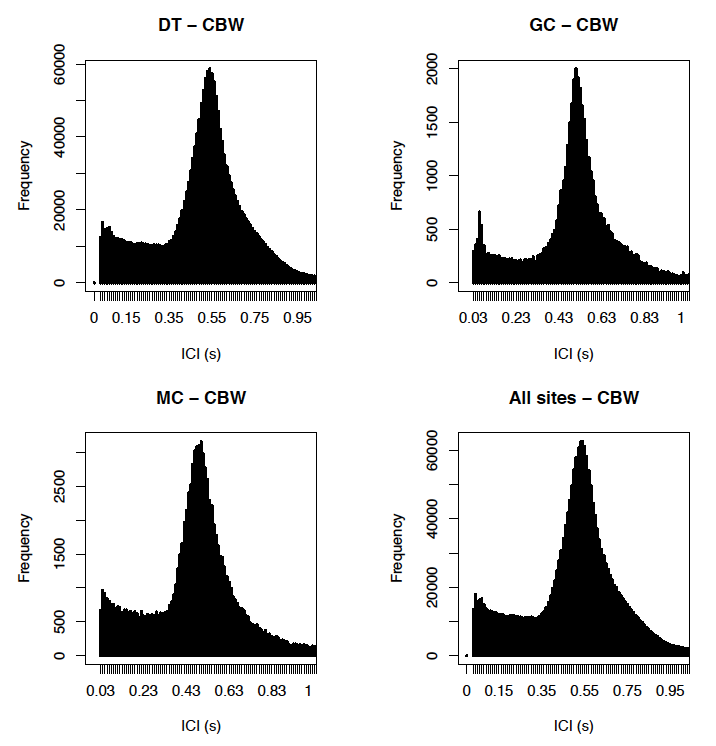

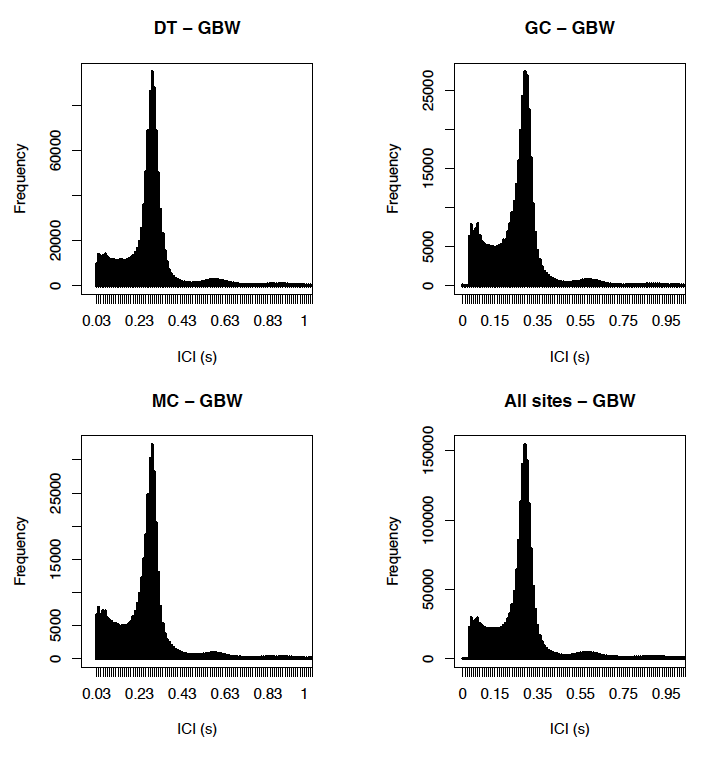


Supplementary Figure S1. Inter-click interval histogram for Cuvier’s beaked whale (CBW on left) and Gervais’ beaked whale (GBW on right) by site (MC, GC and DT) and for all sites.


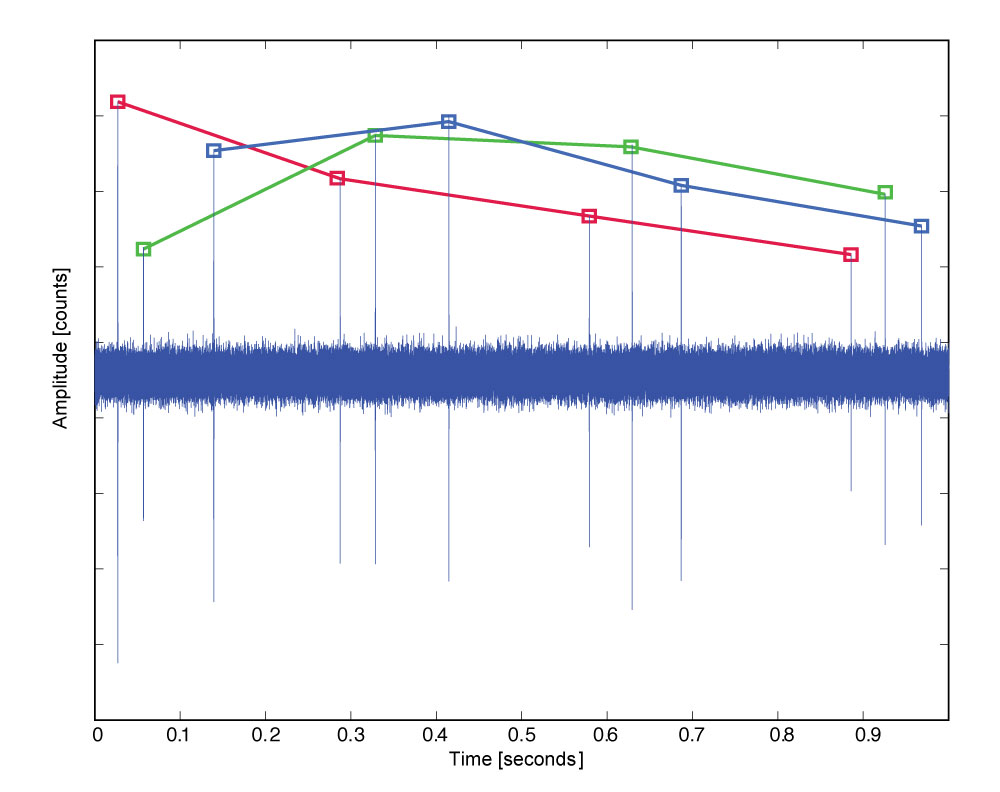


Supplementary Figure S2. Gervais’ beaked whale click sequence detected at the DT site. Individual animal assignment is based on click amplitude and consistent timing. Colored lines join the clicks (boxes) from each of the three animals identified.


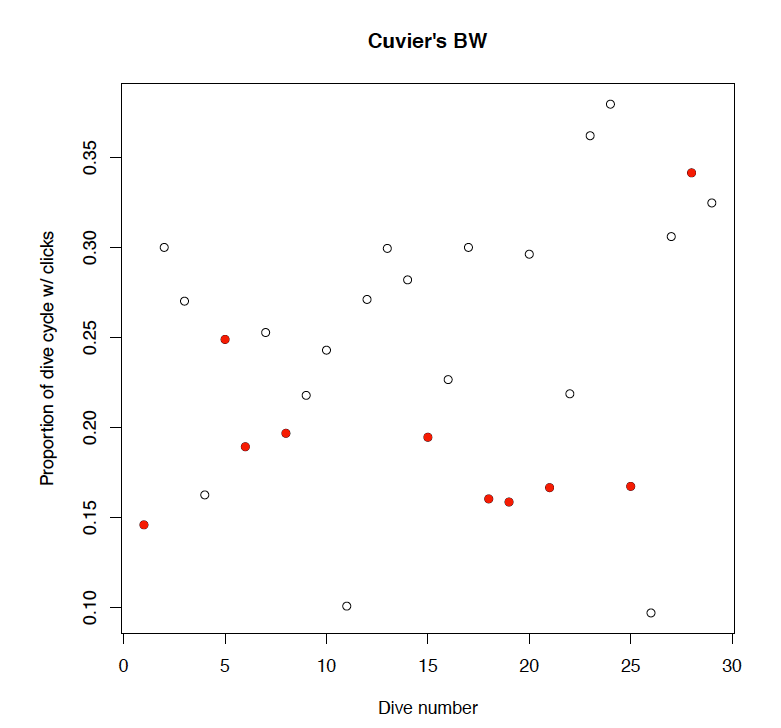

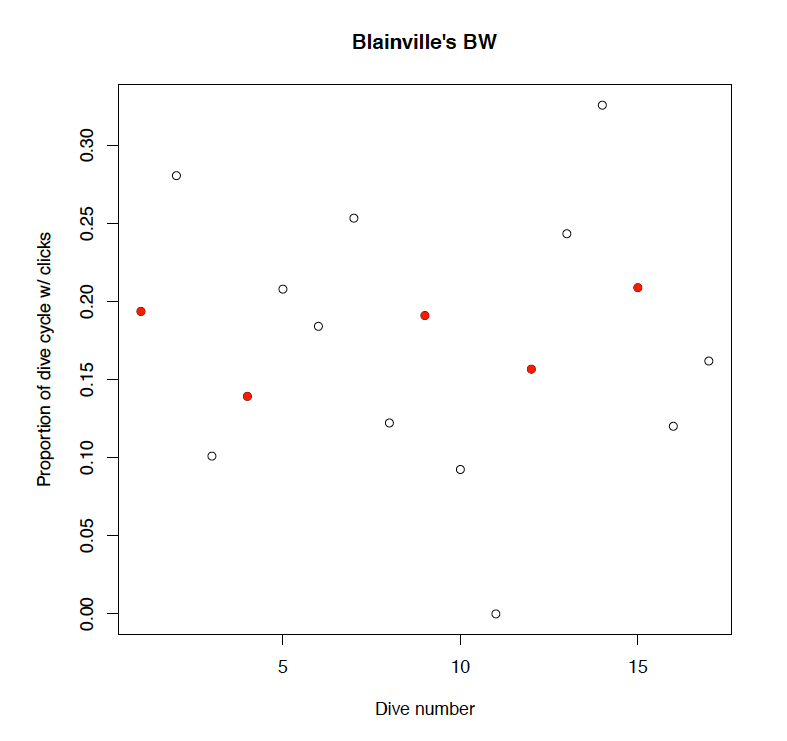


Supplementary Figure S3. Proportion of dive cycle with clicks for Cuvier’s (left) and Blainville’s (right) beaked whales. The red points show the first complete deep dive cycle of each tag deployment, giving a visual assessment of whether a tagging effect may be occurring.


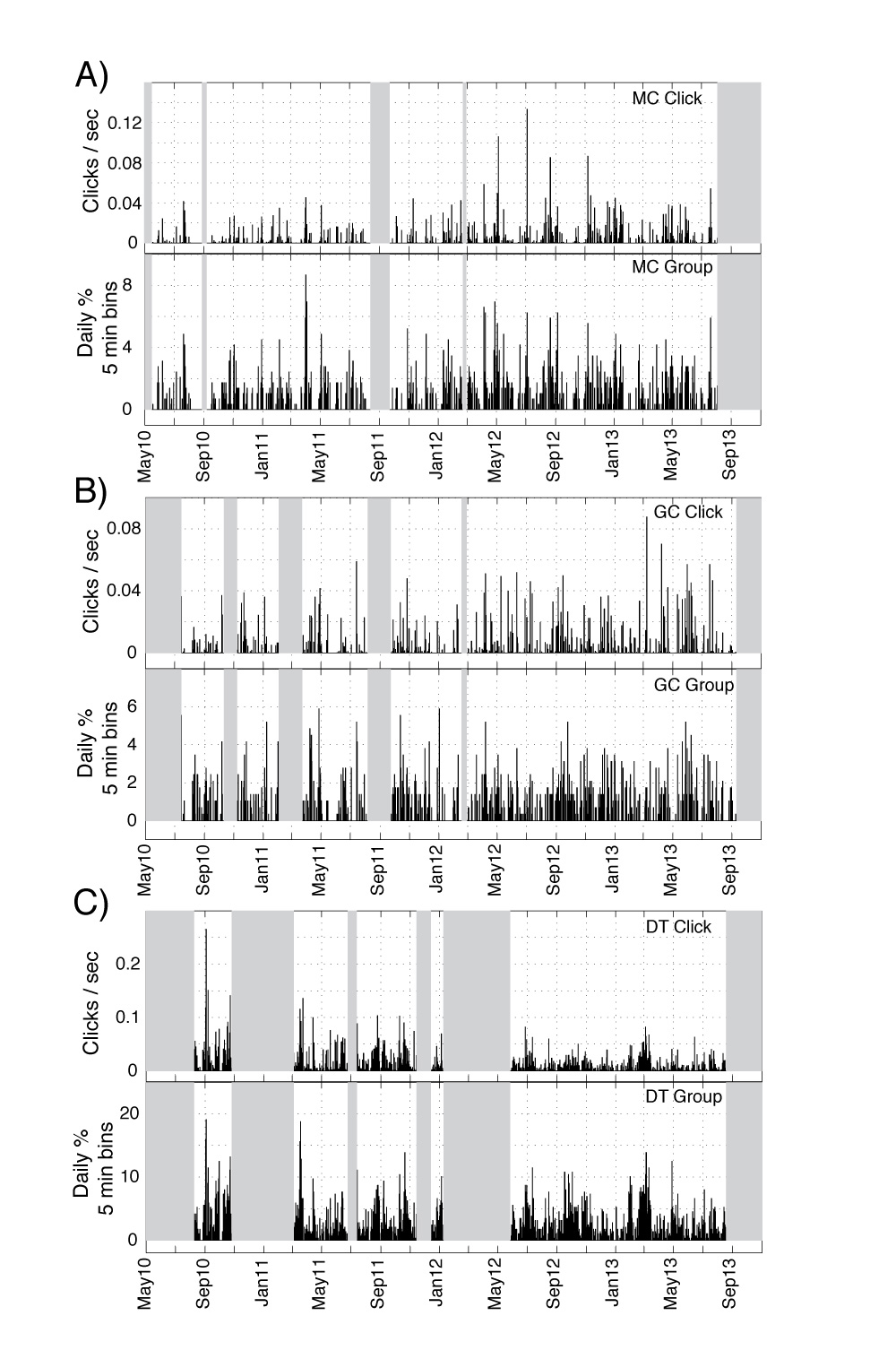


Supplementary Figure S4**.** Gervais’ beaked whale at (A) MC, (B) GC, and (C) DT site. Average click rate daily(Above) . Detections in percentage of 5-min bins daily (Below). Shaded areas lack recording effort.


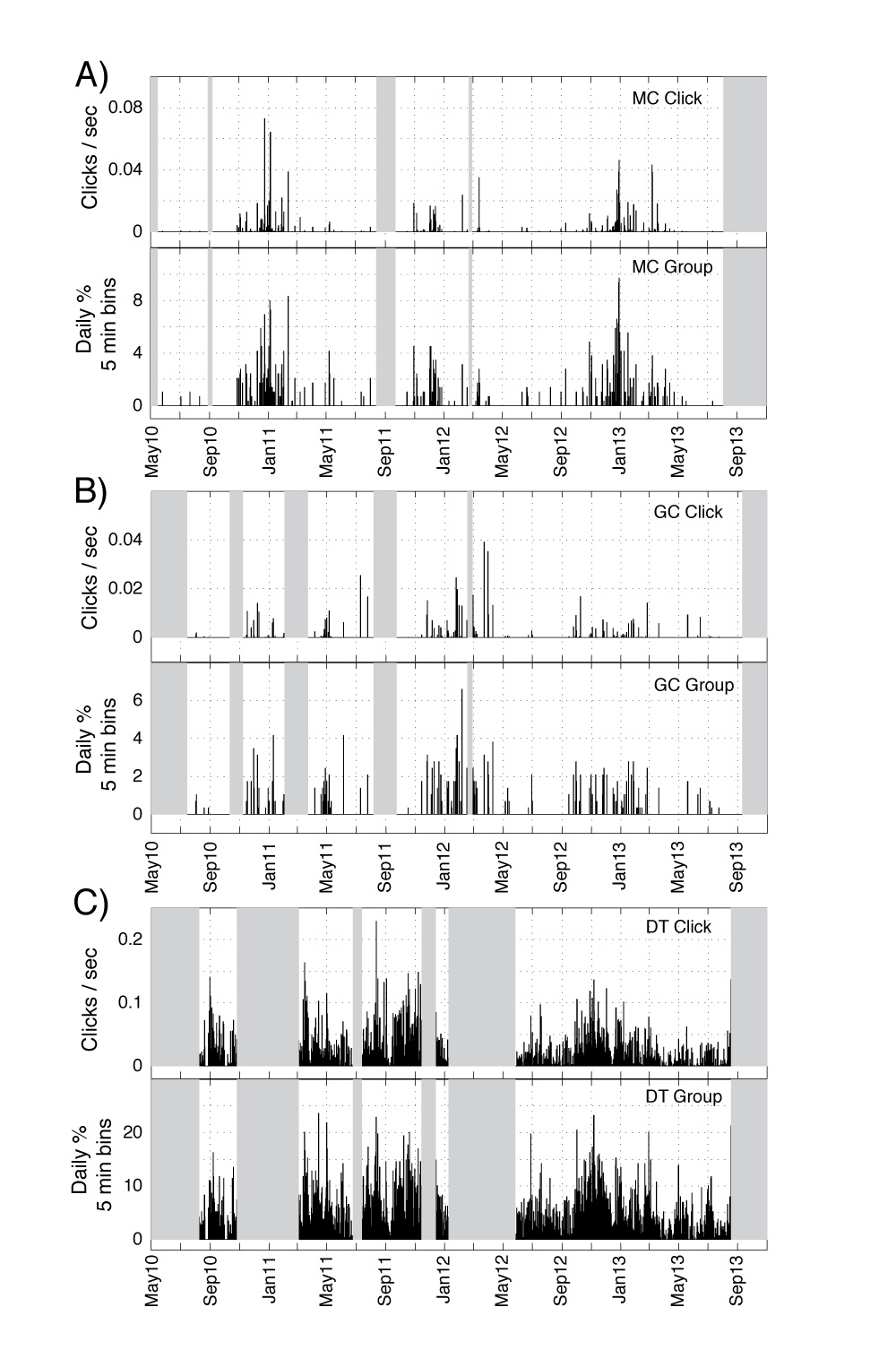


Supplementary Figure S5**.** Cuvier’s beaked whale at (A) MC, (B) GC, and (C) DT site. Average click rate daily(Above) . Detections in percentage of 5-min bins daily (Below). Shaded areas lack recording effort.

*Supplementary Table S1. Modal inter-click interval (ICI) for Cuvier’s, Gervais’, Blainville’s and BWG beaked whale at GOM recording sites, MC, GC and DT.*

| **Site** | **Cuvier’s** | **Gervais’** | **Blainville’s** | **BWG** |
| --- | --- | --- | --- | --- |
|  | **ICI (s)** | **ICI (s)** | **ICI (s)** | **ICI (s)** |
| **MC** | *0.50* | *0.29* | *--* | *0.14* |
| **GC** | *0.52* | *0.29* | *0.32* | *0.14* |
| **DT** | *0.53* | *0.29* | *--* | *0.17* |

*Supplementary Table S2*. HARP deployment periods and locations. Site designations are: MC = Mississippi Canyon, GC = Green Canyon, DT = Dry Tortugas.*

| Site | ID | Start Date  MM/DD/YY | End Date  MM/DD/YY | Recording Duration (Days) | Deployment Long. W | Deployment Lat. N | Deployment Depth (m) |
| --- | --- | --- | --- | --- | --- | --- | --- |
| MC | 01 | 5/16/10 | 8/28/10 | 104.766 | 88-27.927 | 28-50.746 | 980 |
| MC | 02 | 9/7/10 | 12/19/10 | 103.774 | 88-27.907 | 28-50.771 | 980 |
| MC | 03 | 12/20/10 | 3/21/11 | 91.201 | 88-27.909 | 28-50.775 | 980 |
| MC | 04 | 3/22/11 | 8/13/11 | 144.467 | 88-27.946 | 28-50.775 | 980 |
| MC | 05 | 9/22/11 | 2/21/12 | 152.067 | 88-27.991 | 28-50.797 | 980 |
| MC | 06 | 2/28/12 | 12/11/12 | 286.868 | 88-28.041 | 28-50.853 | 980 |
| MC | 07 | 12/11/12 | 8/3/13 | 231.734^#^ | 88-28.059 | 28-50.781 | 980 |
| GC | 01 | 7/15/10 | 10/11/10 | 88.828 | 91-10.010 | 27-33.470 | 1115 |
| GC | 02 | 11/8/10 | 2/2/11 | 86.595 | 91-10.014 | 27-33.466 | 1160 |
| GC | 03 | 3/23/11 | 8/6/11 | 136.131 | 91-10.073 | 27-33.424 | 1100 |
| GC | 04 | 9/23/11 | 2/17/12 | 146.649 | 91-10.060 | 27-33.426 | 1100 |
| GC | 05 | 2/28/12 | 12/12/12 | 288.657 | 91-10.562 | 27-33.440 | 1100 |
| GC | 06 | 12/13/12 | 9/10/13 | 271.135 | 91-10.092 | 27-33.347 | 1100 |
| DT | 01 | 8/9/10 | 10/26/10 | 78.421 | 84-38.251 | 25-31.911 | 1320 |
| DT | 02 | 3/4/11 | 6/24/11 | 112.352 | 84-38.251 | 25-31.911 | 1320 |
| DT | 03 | 7/13/11 | 11/14/11 | 124.421 | 84-38.262 | 25-31.859 | 1300 |
| DT | 04 | 12/14/11 | 1/9/12 | 26.205 | 84-38.265 | 25-31.867 | 1300 |
| DT | 05 | 5/27/12 | 12/7/12 | 193.556 | 84-38.041 | 25-31.938 | 1300 |
| DT | 06 | 12/7/12 | 8/18/13 | 253.214 | 84-38.046 | 25-31.941 | 1300 |

* Two additional monitoring sites on the continental shelf did not yield detections of beaked whales (Main Pass = 29-15.204 N, 88-17.753 W, depth 86 m; and DeSoto Canyon = 29-03.134 N, 86-05.773 W, depth 268 m). ^#^Accounts for recording gap.
